# Rheological properties and compressive properties of alkali-activated slag-fly ash geopolymer fluid solidified soil

**DOI:** 10.1371/journal.pone.0350229

**Published:** 2026-07-10

**Authors:** Chunxiao Qi, Boshen Wang, AnHui Chen, Haiting Zhang, Shuai Pang

**Affiliations:** 1 China Construction Sixth Bureau Civil Engineering Co.Ltd., Tianjin, China; 2 College of Water Resources and Architectural Engineering, Northwest A&F University, Yangling, China; University of Greenwich, UNITED KINGDOM OF GREAT BRITAIN AND NORTHERN IRELAND

## Abstract

To study the influence of the content of cementitious materials, water-solid ratio, fiber content and NaOH content on the rheological properties and compressive properties of geopolymer fluid solidified soil, and to reveal the influence mechanism of different factors on geopolymer fluid solidified soil (GFSS). Geopolymers prepared from slag and fly ash were mixed into aeolian soil as cementing materials, and the rheological parameters of GFSS were tested by MCR rheometer, and its compressive strength was tested after curing for 28 days, and its micro-morphology was obtained by SEM. When the content of cementing material is increased from 8% to 16%, the fluidity of GFSS decreased by 62.01 mm and the compressive strength increases by 121.52%. When the water-solid ratio increased from 0.26 to 0.34, the fluidity increased by 81.79%, and the compressive strength first increased and then decreased, reaching the peak when the water-solid ratio was 0.30. With the increase of fiber content, the fluidity will decrease, and the compressive strength will also increase first and then decrease. When the fiber content is 5‰, the fiber will form a stable three-dimensional network structure, and the compressive strength will reach 1.19MPa. With the increase of NaOH content, the fluidity of slurry decreases, the system structure becomes denser and the mechanical properties are significantly improved. When the proportion of NaOH is 6%, cementing material is 12%, water-solid ratio is 0.30, and fiber content is 5‰, the comprehensive performance of GFSS is good, which can meet the application requirements of secondary excavation and backfill engineering. The research results are of great significance for improving the design standard of solidified soil backfill engineering, increasing the service life of solidified soil backfill engineering and reducing the disaster of pavement collapse in seasonal frozen soil areas.

## 1 Introduction

There is a widespread demand for backfilling in areas such as basement foundation trenches, municipal utility trenches, road collapse zones, and karst caves. However, backfilling is a concealed construction process, making quality control challenging. These areas typically feature narrow backfilling spaces and considerable depths, imposing strict requirements on backfill material quality [1]. Traditional backfilling methods, such as manual compaction or the use of graded gravel or sandy soil, often require extensive mechanical equipment during compaction, resulting in complex operations and slow construction progress [2]. When backfilling around underground pipelines or structures, excessive compaction vibration may damage adjacent pipelines or structures [3]. Against the backdrop of increasingly complex modern engineering projects and rising environmental requirements, the limitations of traditional backfilling methods have become more apparent. Flowable solidified soil, as a new energy-saving, environmentally friendly, and cost-effective material, offers significant advantages in backfilling applications [4].

Geopolymers are synthesized from aluminosilicate-rich industrial byproducts (e.g., fly ash, slag) [5]. Their production process significantly reduces carbon emissions, aligning with sustainable development principles [6]. Geopolymers exhibit superior mechanical properties, including high compressive strength [7], excellent crack resistance [8], and durability [9], demonstrating broad applicability in engineering fields [10]. In recent years, many comprehensive studies on geopolymer solidified soil show that the formation of geopolymer gel has been verified in stable soil, that is, geopolymer can effectively improve the mechanical strength of soil [11–14]. Some scholars add fiber to controllable low-strength materials to improve their toughness, and good improvement results have been obtained [15–19]. Despite its excellent performance, the long-term stability in complex freeze-thaw environment still needs to be further improved. Previous scholars have improved the frost resistance of geopolymer solidified soil in different ways and achieved good results [20–23]. In projects such as soft soil stabilization and road rehabilitation, flowable solidified soil is highly favored due to its ease of construction and superior filling characteristics [24]. The rheological properties of flowable solidified soil are influenced by multiple factors, such as the curing agent composition and water-to-solid ratio [25], with complex interactions among these factors increasing the intricacy of rheological behavior studies [26]. At present, a lot of work has been done on fluid solidified soil, but it mainly focuses on the mechanical properties and durability of hardened paste, and there are few reports on the rheological properties of fresh paste.

Based on the defects of previous research, this paper prepared GFSS by slag-fly ash, analyzed the rheological properties of GFSS through steady-state shear test system, revealed the evolution characteristics of internal structure of GFSS during curing through microscopic observation, and combined with macroscopic performance research, clarified the influence mechanism of microstructure on mechanical properties. The research results promote the application research of geopolymer in the field of fluid solidified soil, and provide a new idea for the low-carbon and performance improvement of traditional fluid solidified soil, which is in line with the policy goal of “peak carbon dioxide emissions” and “carbon neutrality” and contributes to the low-carbon transformation in the field of building materials.

## 2 Test scheme design

### 2.1 Testing material

**Fig 1:**
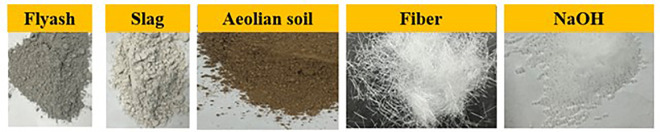
Test raw material diagram.

The test specimens used aeolian soil from western Liaoning as the primary curing material. Basic property tests were conducted on the aeolian soil according to geotechnical testing standards, with the following results: particle specific gravity of 2.63, maximum dry density of 1.61 g/cm^3^, optimum moisture content of 13.4%, liquid limit of 25.53%, plastic limit of 17.19%, and plasticity index of 8.34. The cementitious material was alkali-activated geopolymer. The slag used was Grade S95, purchased from Yixiang New Materials Co., Ltd. in Henan Province, fly ash was sourced from Henan Hengyuan New Materials Co., Ltd., and NaOH was obtained from Liaoning Quanrui Reagent Co., Ltd. The experimental raw materials are shown in Fig 1. The physical property parameters of polypropylene fiber are shown in Table 1.

**Table 1:** Physical properties of polypropylene fiber.

Fibre length /mm	Density/（g·cm^-3^）	Tensile strength/MPa	Elastic modulus/GPa	Melting point/℃	Ignition point/℃	Fracture elongation /%	Dispersibility
9	0.91	≥350	≥3.5	>160	590	≥10	Fabulous

**Fig 2:**
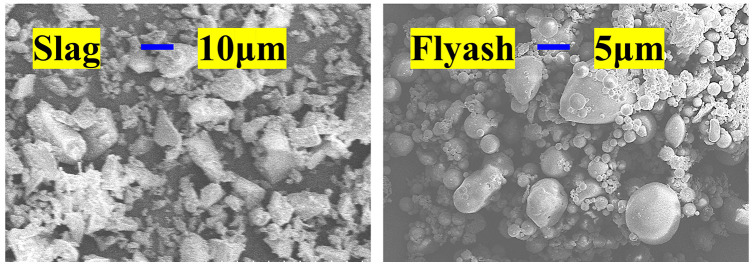
SEM diagram of cementitious materials.

The SEM images of slag and fly ash are shown in Fig 2. As can be seen from Fig 2, the slag particles are irregular block shapes, while the fly ash particles are spherical.

The test results of chemical composition of slag and fly ash are shown in Table 2. The chemical composition test results of slag and fly ash are shown in Table 2. The fly ash used in the test is Class I fly ash, which meets the Chinese national standard GB / T 1596–2017. According to its chemical composition (as shown in Table 2), the fly ash belongs to the typical F grade fly ash according to the international ASTM C618 standard.

**Table 2:** Chemical composition of raw materials.

Material	CaO	Fe_2_O_3_	Al_2_O_3_	SiO_2_	MgO	SO_3_	K_2_O	Na_2_O
Slag	42.15	0.41	12.88	33.51	5.98	–	0.21	0.28
Flyash	4.15	3.65	24.99	59.11	0.42	0.35	1.15	0.99

### 2.2 Test scheme

This study primarily investigates the effects of binder content, water-to-solid ratio, fiber dosage, and NaOH content on the rheological properties and compressive strength of GFSS. Based on previous research by scholars [27,28], experimental design is shown in Table 3.

**Table 3:** Test plan.

Number	Cementitious material content	Water-solid ratio	Fiber content /‰	NaOH content /%
A1	8%	0.30	5	4
A2	10%			
A3	12%			
A4	14%			
A5	16%			
B1	12%	0.26	5	4
B2		0.28		
B3		0.30		
B4		0.32		
B5		0.34		
C1	12%	0.30	3	4
C2			4	
C3			5	
C4			6	
C5			7	
D1	12%	0.30	5	2
D2				4
D3				6
D4				8
D5				10

**Fig 3:**
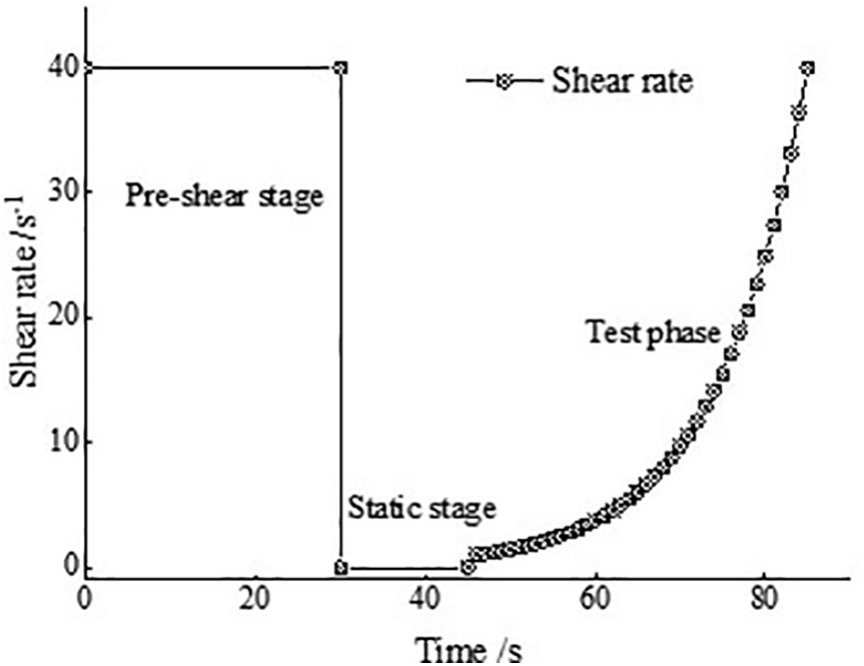
Rheological loading modemode.

In the test scheme, the content of cementitious material is the mass ratio of cementitious material to aeolian soil, the water-solid ratio is the ratio of water mass to all solid mass, the fiber content is the mass ratio of fiber mass to aeolian soil. The dosage of NaOH activator (such as 10%) in this study is based on the total mass of cementitious materials (slag and fly ash), not on the total mass of treated soil. Because the maximum design dosage of cementing material in aeolian soil in this experiment is 16%, the actual proportion of NaOH in the total mass of aeolian soil after treatment is only about 1.6% at this extreme ratio. This relatively low actual soil content is an important prerequisite for the subsequent evaluation of the economy and environmental safety of the curing system. In this study, the mass ratio of blast furnace slag (BFS) to fly ash (FA) is fixed at 1:1. The selection of this ratio is mainly based on the Synergistic effect: the high reactivity of slag can ensure the early mechanical strength of the system, while the ball effect of fly ash can effectively improve the working performance of the slurry and inhibit the later shrinkage. Through the ratio of 1:1, the aim is to balance the setting time, fluidity and comprehensive mechanical properties of the material, so as to obtain a matrix with balanced properties [29]. During the test, the samples were prepared according to the ' Technical Standard for Engineering Application of Premixed Fluidized Solidified Soil ‘. In the process of sample preparation, to avoid the influence of temperature on the properties of cementitious materials, NaOH was weighed according to the test scheme before sample preparation, added into water and stirred evenly, and stood for 12 hours after NaOH was completely dissolved. Slag, fly ash, aeolian soil and polypropylene fiber are weighed according to the proportion of solid materials in the test scheme, and then they are respectively weighed and poured into a mortar mixer for mixing and slowly stirring for 2 min. After stirring evenly, NaOH solution is poured into dry mixed materials, and stirring is continued for 3 min until the mixture is in a uniform state. Subsequently, fluidity test and steady shear test were carried out. The steady-state shear loading mode is shown in Fig 3.

After completing the rheological tests, the GFSS was poured into 70.7 mm × 70.7 mm × 70.7 mm cubic molds, compacted on a vibration table, demolded after curing at room temperature, and then placed in a constant temperature and humidity curing chamber (temperature 20 ± 2°C, humidity approximately 90%) for 28 days of curing. Three parallel specimens were prepared for each mix proportion, totaling 60 specimens.

**Fig 4:**
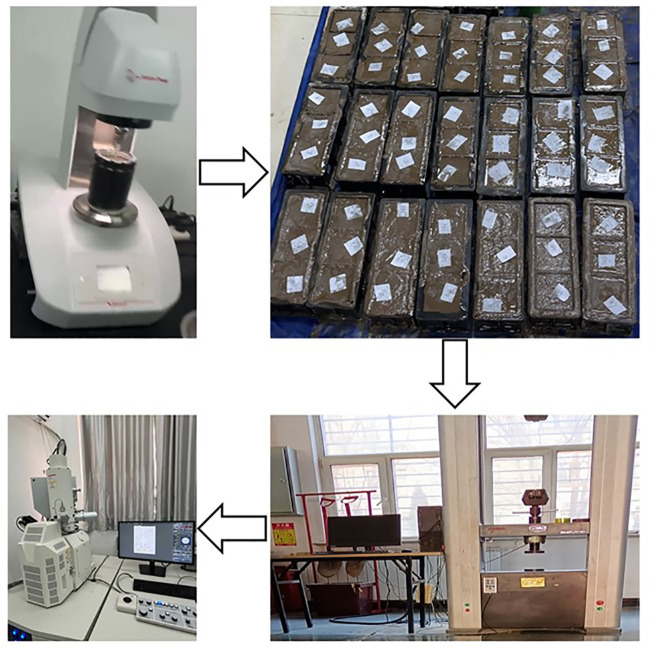
Test processprocess.

After curing, unconfined compressive strength tests were conducted using a WDW-100E microcomputer -controlled electronic universal testing machine with a loading rate of 1 mm/min (GB/T 39489−2020). Following the test, selected samples were cut into 5mm^3^ cubes and subjected to microstructural analysis using a Hitachi, SU5000 Compact field emission scanning electron microscope. The testing procedure is illustrated in Fig 4. This article does not include any studies involving humans or animals.

## 3 Analysis of test results

### 3.1 Test results of flow spread

**Fig 5:**
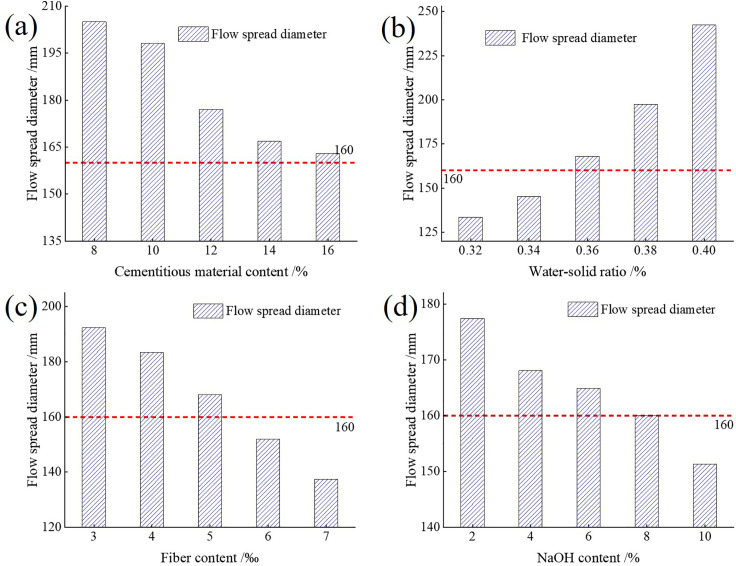
Flow spread of samples under different conditions. (a) Different dosage of cementitious materials. (b) Different water-solid ratio. (c) Different fiber content. (d) Different NaOH content.

The GFSS with different blending schemes was tested for flow expansion, and the test results are shown in Fig 5.

As can be seen from Fig 5(a), the flow spread of GFSS gradually decreases with the increase of the content of cementing material, and when the content of cementing material increases from 8% to 16%, the flow spread of GFSS decreases from 205 mm to 163 mm. This phenomenon is mainly caused by the combined action of chemical reaction kinetics and physical rheological effect. Firstly, the specific surface area of slag and fly ash is large, and the increase of the dosage significantly increases the total specific surface area of the system, which consumes a lot of free water for wrapping particles, resulting in an increase in friction resistance between particles. Secondly, more importantly, with the increase of the concentration of cementitious materials, the reaction rate of alkali excitation is significantly accelerated. Slag contains a lot of active calcium. Under the action of alkaline activator, the glass network structure of slag and fly ash is rapidly depolymerized. When the content of cementing material is increased from 8% to 16%, many Ca^2+^, Si^4+^ and Al^3+^ ions will be dissolved in a very short time, and many hydrated aluminosilicates gel products will be generated quickly. The formation of these early gels not only transformed the free water in the system into bound water but also promoted the rapid formation of flocculation network structure in the slurry, which significantly improved the yield stress and viscosity of the system, and finally led to the decrease of macro-flow diffusion ability.

Fig 5(b) demonstrates that the flow spread diameter of GFSS progressively increased with higher water-to-solid ratios. As the water-to-solid ratio rose from 0.26 to 0.34, the flow spread diameter expanded from 133 mm to 242 mm, representing a 109 mm increase. The increased water-to-solid ratio indicates more water per unit mass of solid materials. This additional water coats solid particles, reducing direct particle contact and improving particle dispersion. At lower ratios, particle accumulation or bridging effects occur, restricting slurry fluidity. Higher ratios mitigate this accumulation as water fills interparticle voids, creating a more homogeneous mixture that prevents localized particle aggregation. Furthermore, enhanced water coverage on particle surfaces improves liquid-solid interface wetting, reducing interparticle friction. This facilitates particle slippage and flow, ultimately increasing the spread diameter. The water film acts as a lubricant between particles, enabling easier rearrangement and movement under gravitational forces during the flow test. The improved particle mobility directly correlates with the observed expansion of the flow spread diameter.

Fig 5(c) shows that the flow spread diameter of GFSS gradually decreased with increasing fiber content. When the fiber content was 3‰, the flow spread diameter of GFSS was 192 mm. As the fiber content increased to 7‰, the flow spread diameter decreased by 28.65% to 137 mm. As a solid phase, fibers form a complex network structure in the slurry that hinders free flow. At low fiber content, fibers have limited effect on enhancing the slurry structure, resulting in minimal impact on flow spread diameter. When fiber content reaches 5‰, the presence of fibers reduces relative slippage between particles and increases flow resistance, leading to a significant decrease in spread diameter.

As can be seen from Fig 5(d), with the increase of NaOH content, the flow spread of GFSS gradually decreases. When NaOH content increased from 2% to 10%, the flow spread of GFSS decreased by 14.69%. This is because the increase of NaOH content will increase the dissolution rate of active silicon (Si) and aluminum (Al) in slag and fly ash, thus rapidly generating more geopolymer gels, which will significantly increase the viscosity of the slurry, which will hinder the relative slip of particles after the increase of viscosity, thus significantly reducing the flow spread of the slurry.

### 3.2 Rheological performance test results

**Fig 6:**
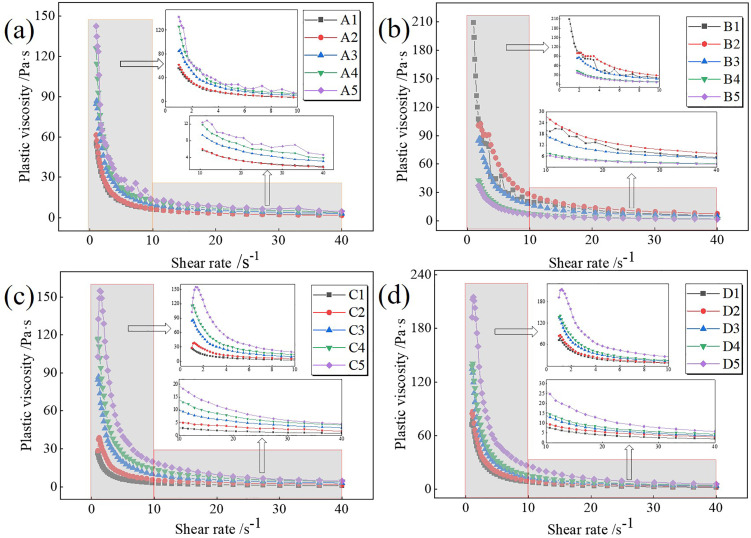
Relationship between sample viscosity and shear rate under different conditions. (a) Different dosage of cementitious materials. (b) Different water-solid ratio. (c) Different fiber content. (d) Different NaOH content.

The relationship between GFSS viscosity and shear rate under different conditions is shown in Fig 6.

As can be seen from Fig 6, at low shear rates (0-10s^-1^), the slurry exhibits a higher viscosity because there is a strong interaction force between solid particles, molecular chains or aggregates. As the shear rate increases, the viscosity value of GFSS drops rapidly. This is because during the process of increasing shear rate, the particles or molecular chains in the GFSS slurry begin to rearrange or depolymerize under the action of external shear force. The originally disordered particle structure will gradually become orderly, reducing friction and resistance between particles. At the same time, the aggregates are gradually destroyed or dispersed under the action of shear force, and the slurry becomes easier to flow, manifesting as a decrease in viscosity. When the shear rate further increases to a certain level (10s-1 ~ 40s-1), the viscosity gradually stabilizes. This is because at high shear rate, the interaction force between particles becomes smaller, the rearrangement or depolymerization of particles or molecular chains has been basically completed, and the microstructure inside the fluid has adapted to the flow state of high shear rate. At this time, the flow resistance of the GFSS slurry at a high shear rate tends to balance and the viscosity remains at a low constant value.

**Fig 7:**
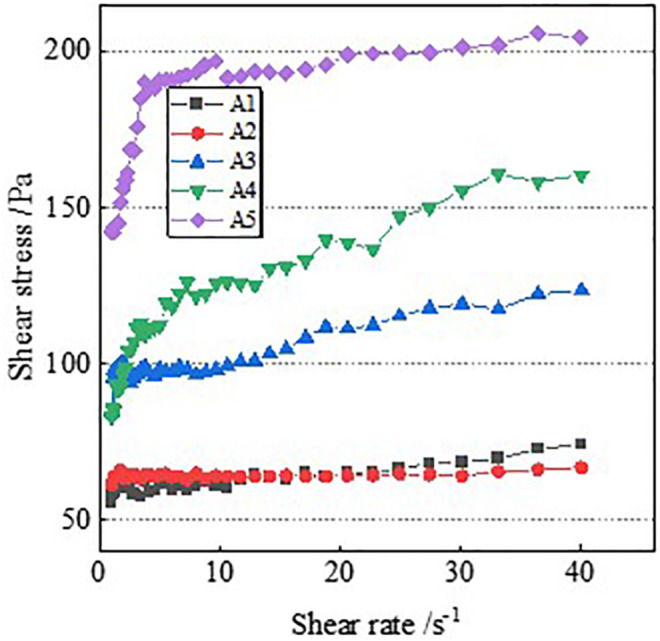
Relationship between shear rate and shear stress of samples with different cementitious materials content.

The relationship between GFSS shear stress and shear rate of different gelling materials is shown in Fig 7.

As shown in Fig 7, the rheological test further shows that the shear stress of GFSS gradually increases with the increase of the content of slag-fly ash-based cementitious materials, which is highly consistent with the reduction law of macro-flow spread. From the point of view of chemical kinetics, the increase of the content of cementitious materials accelerates the early geological polymerization and hydration reaction, and a large number of C-(A)-S-H and N-A-S-H gels generated rapidly in the system form a strong bridge and cross-link between soil particles, and a micro-network structure with high shear resistance is constructed. Secondly, a large amount of free water is consumed and converted into gel-bound water, which leads to the thinning of lubricating water film on the surface of particles, and the direct contact and mechanical friction between solid particles are significantly intensified. The superposition of these physical and chemical changes greatly increases the energy required to destroy the initial structure of the slurry, which is manifested as a significant increase in GFSS shear stress.

**Fig 8:**
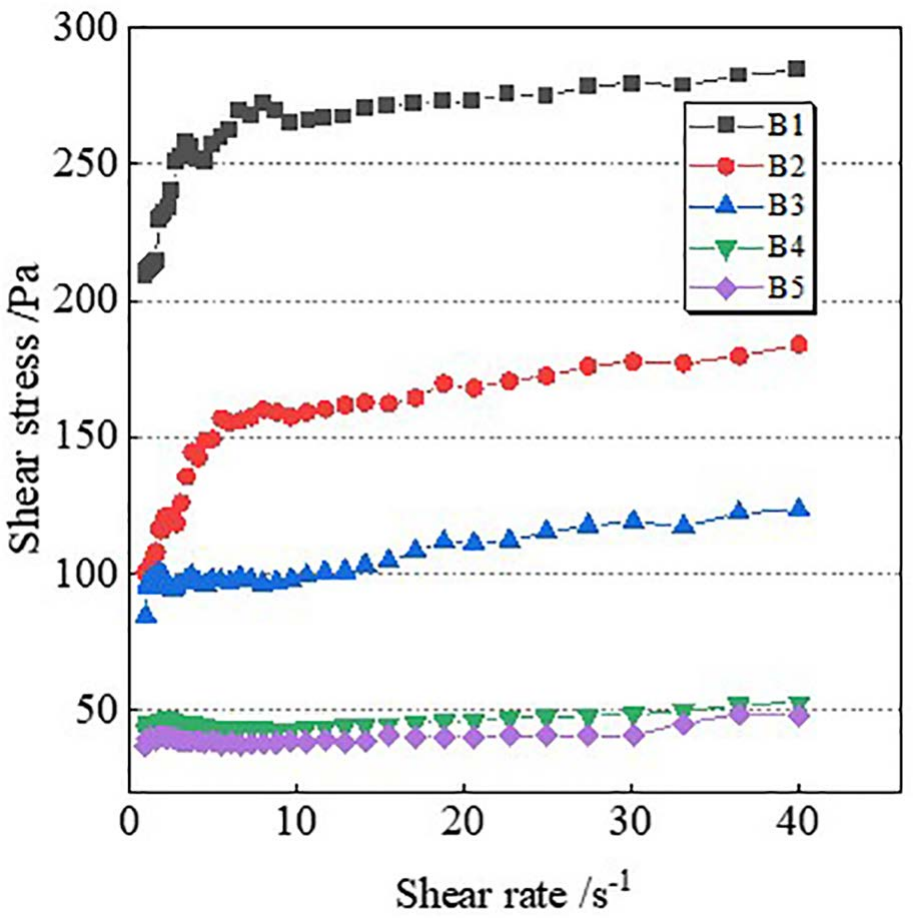
Relationship between shear stress and shear rate of samples with different water-solid ratio.

The relationship between shear stress and shear rate of GFSS with different water-solid ratios is shown in Fig 8.

**Fig 9:**
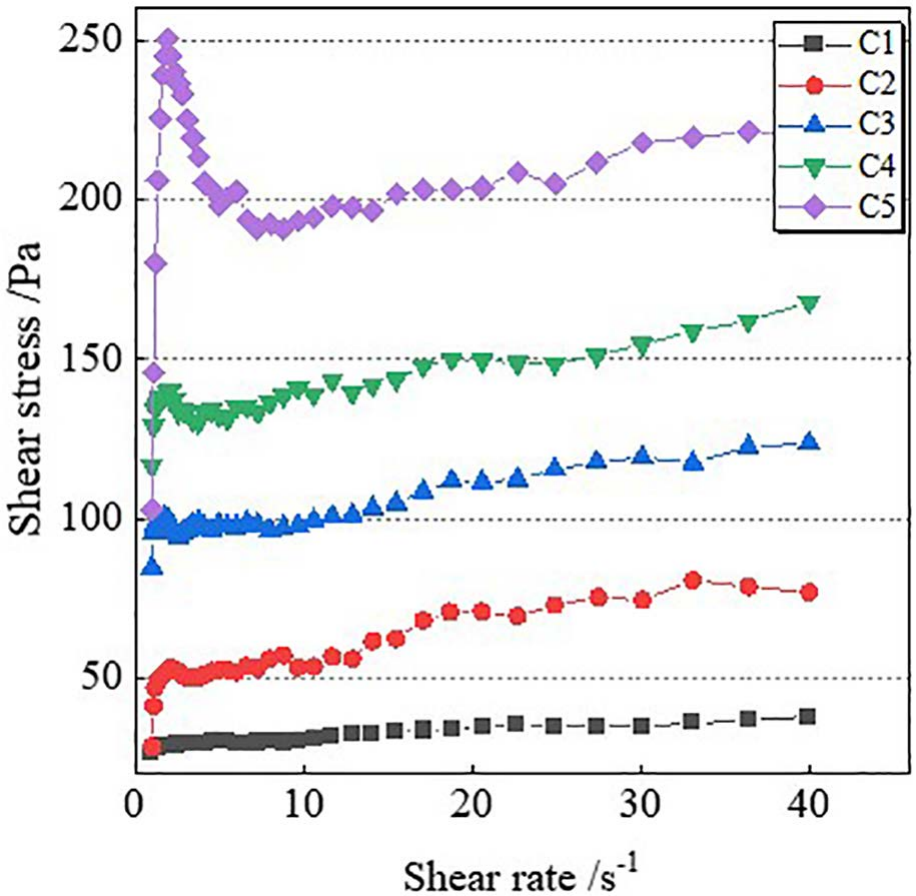
Relationship between shear stress and shear rate of samples with different fiber contentcontent.

As shown in Fig 8, the shear stress of GFSS gradually decreases with the increase of water-solid ratio. With the increase of water-solid ratio, the water in the system is more. As a solvent, the lubrication between the particles is strengthened, and the particles are more likely to slide. The increase of water reduces the viscosity of the material, thus reducing the friction and adhesion between the particles inside the material. Therefore, the increase of water-solid ratio will gradually reduce the shear stress of GFSS. The relationship between shear stress and shear rate of GFSS with different fiber content is shown in Fig 9.

As shown in Fig 9, with the increase of fiber content, the shear stress of GFSS increases gradually. When the fiber content is low (3‰ ~ 4‰), the fiber and the geopolymer matrix are intertwined to form an enhanced three-dimensional network structure, thereby increasing the shear resistance of the material and increasing the shear stress. When the fiber content is 5‰, an appropriate amount of fiber can form enough connection points and staggered structures to provide more shear resistance sources. In this case, the strength and shear resistance of the material are significantly improved, resulting in an increase in shear stress. When the fiber content is too high (6‰ ~ 7‰), the fibers accumulate or agglomerate during the GFSS rheological test, resulting in most of the fibers gathering near the rotor. This phenomenon causes the fibers to hinder the flow inside the matrix, increasing the internal friction and flow resistance, and increasing the shear stress.

**Fig 10:**
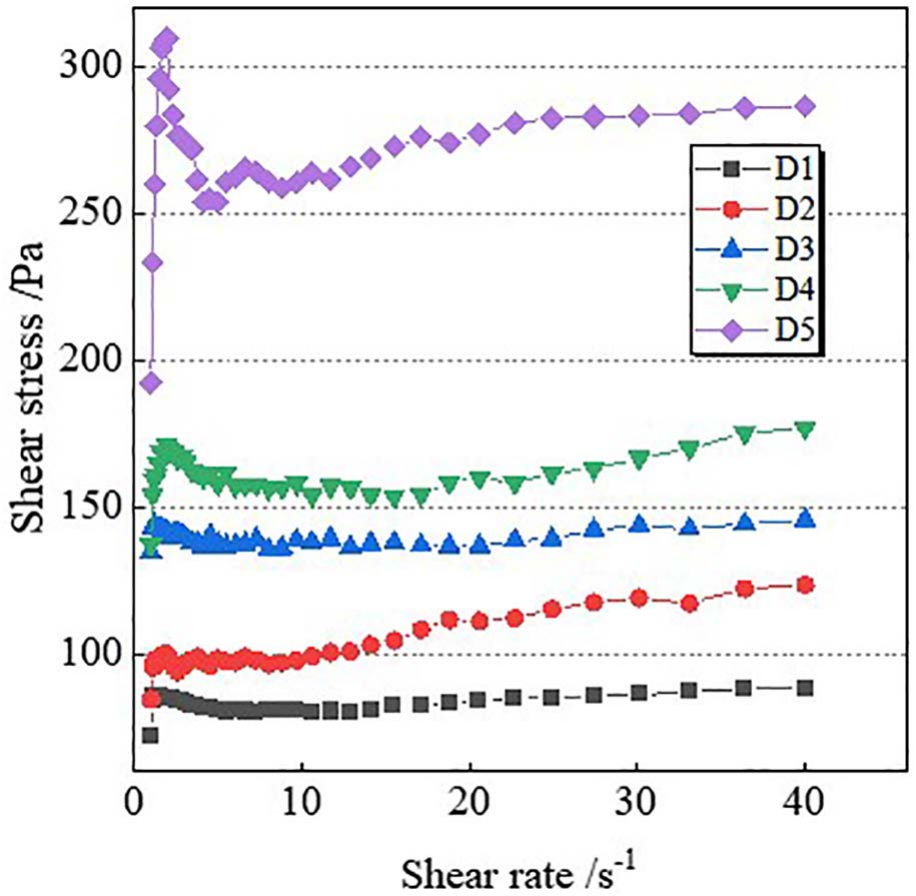
Relationship between shear rate and shear stress of samples with different sodium hydroxide content.

The relationship between shear stress and shear rate of GFSS with different NaOH content is shown in Fig 10.

As shown in Fig 10, with the increase of NaOH content, the shear stress of GFSS increases gradually. This is because NaOH is used as an alkali activator in the GFSS system. When the content of NaOH increases, the alkalinity (pH value) and ion concentration in the GFSS system increase, which not only accelerates the reaction, but also enhances the cementitious properties of the matrix, so that the material exhibits higher shear resistance.

From Figs 7–10, with the increase of shear rate, the shear stress curve is roughly divided into two regions, namely stress overshoot region and shear thinning region. The shear rate of 0 ~ 10s-1 belongs to the stress overshoot zone, which is mainly characterized by a fluid stress hysteresis caused by the internal friction of the fluid when the external force is disturbed. When the shear rate is 10s-1 ~ 40s-1, it belongs to the shear thinning zone, which mainly shows that the increase of shear stress becomes smaller and tends to be stable with the increase of shear rate. Because the shear thinning zone (shear rate is 10 ~ 40s^-1^) accounts for the largest proportion in the shear process and the slope of the shear thinning zone is roughly the same as that of the composite Bingham fluid characteristics, the shear thinning zone is selected as the research object of the rheological properties of the fiber reinforced fluid solidified soil. The Bingham rheological model is used to fit the shear thinning zone, and the Bingham rheological model is shown in Formula 1.


$$\begin{array}{c} \tau =\tau_{\text{0}}+\eta \dot{\gamma } \end{array}$$ (1)

In the formula: τ is the shear stress, τ0 is the yield stress, η is the plastic viscosity, $\dot{\gamma }$ is the shear rate.

According to Formula 1, the rheological curves of GFSS slurry under different conditions were fitted respectively, and the results are shown in Table 4.

**Table 4:** Fitting results of Bingham model under different factors.

Test number	Yield stress $\tau_{0}$/pa	Plastic viscosity η/pa·s	Fitting equation	R2
1	57.50	0.39	τ=57.50+0.39˙	0.93
2	62.61	0.09	τ=62.61+0.09˙	0.76
3	92.49	0.84	τ=92.49+0.84˙	0.94
4	110.59	1.37	τ=110.59+1.37˙	0.95
5	186.82	0.48	τ=186.82+0.48˙	0.95
6	261.07	0.60	τ=261.07+0.60˙	0.97
7	151.24	0.83	τ=151.24+0.83˙	0.97
8	92.49	0.84	τ=92.49+0.84˙	0.94
9	40.17	0.31	τ=40.17+0.31˙	0.98
10	34.70	0.31	τ=34.70+0.31	0.79
11	29.82	0.20	τ=29.82+0.20˙	0.89
12	49.71	0.84	τ=49.71+0.84˙	0.83
13	92.50	0.84	τ=92.50+0.84˙	0.94
14	130.56	0.86	τ=130.56+0.86˙	0.93
15	185.24	0.96	τ=185.24+0.96˙	0.94
16	77.33	0.30	τ=77.33+0.30˙	0.97
17	92.50	0.84	τ=92.50+0.84˙	0.94
18	133.13	0.30	τ=133.13+0.30	0.79
19	143.88	0.78	τ=143.88+0.78˙	0.91
20	257.92	0.82	τ=257.92+0.82˙	0.85

From Table 3, the corrected complex correlation coefficient r of the curve fitted by Bingham model is greater than 0.75, and the fitting effect is good, which shows that Bingham model has accurate prediction ability for GFSS slurry. According to Table 3, the yield stress of GFSS increases gradually with the increase of the content of cementing material. When the content of cementing material is 8%, the yield stress of the sample is 57.50pa, and when the content of cementing material is 16%, the yield stress of GFSS slurry increases to 186.82pa, an increase of 129.32pa. With the increase of the water-solid ratio, the yield stress of GFSS gradually decreases. As the water-solid ratio increases, the amount of water in the slurry increases, and the liquid phase forms a more uniform distribution between the particles, reducing the friction resistance between the particles, and causing the slurry to exhibit lower yield stress. At the same time, moisture plays a dilution and isolation between the surface of the soil particles and the gelled material, weakening the interface bonding strength, reducing the cohesion between the particles, and thus reducing the yield stress. While the fiber dosage increases, the yield stress of GFSS gradually increases, because as the fiber dosage increases, the fibers in the slurry, soil particles and gelling materials form a denser three-dimensional framework structure. The presence of fibers makes the connection between particles more stable, reduces the relative sliding between particles, and the overall structure of the slurry is more stable, improving the ability of the slurry to resist shear. With the increase of NaOH dosage, the yield stress of the GFSS slurry increases significantly. This is because after the increase of NaOH dosage, NaOH increases the alkaline environment of the slurry, enhances the solubility of aluminum-silicon raw materials, and allows more silicon-oxygen tetrahedron (SiO₄) and aluminum-oxygen tetrahedron (AlO₄) units to dissolve into the liquid phase. This dissolution enhances the initial viscous network structure in the slurry, thereby increasing yield stress. In addition, an increase in NaOH concentration will significantly increase the viscosity of the liquid phase of the slurry. The increase in liquid phase viscosity limits the relative movement of particles in the slurry, causing the slurry to exhibit higher yield stress.

## 4 Analysis of unconfined compressive strength

**Fig 11:**
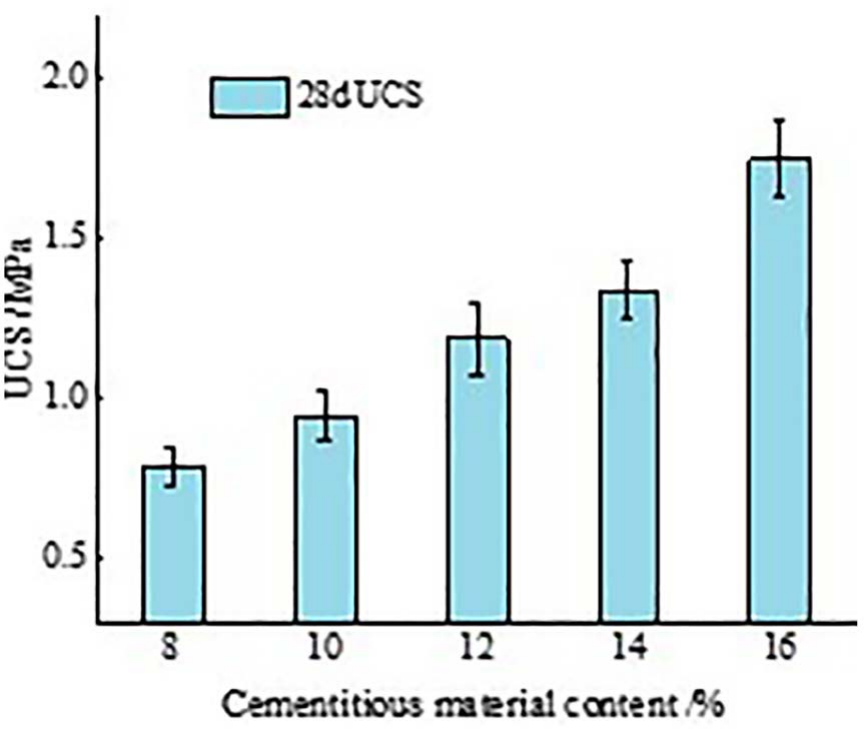
Compressive strength of samples with different cementitious materials content.

The unconfined compressive strength of GFSS with different cementitious material content was tested, and the test results are shown in Fig 11.

**Fig 12:**
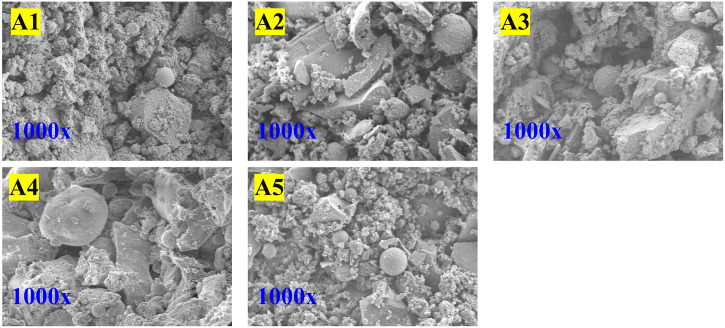
Micro-morphology of samples with different cementitious materials content.

From Fig 11, with the increase of cementitious material content, the compressive strength of GFSS sample at 28 d gradually increases. When the cementitious material content is 16%, the compressive strength of the sample reaches a local maximum of 1.75 MPa. This is because the C-A-S-H and N-A-S-H gel generated by the geopolymer reaction is a three-dimensional network structure material with high adhesion, which can significantly improve the strength of the material. Too low content is not enough to generate enough gel, resulting in limited strength improvement. Combined with Fig 12, the microstructure inside the GFSS sample is denser and more stable after the content is increased, and the C-A-S-H and N-A-S-H gel is filled in the pores of the solidified soil and plays a ' skeleton ' role. At the same time, the cementitious material also plays a bonding role between particles. The bonding and filling effects of the gel when the sample is loaded effectively inhibit the generation and expansion of microcracks and improve the compressive strength of the material.

**Fig 13:**
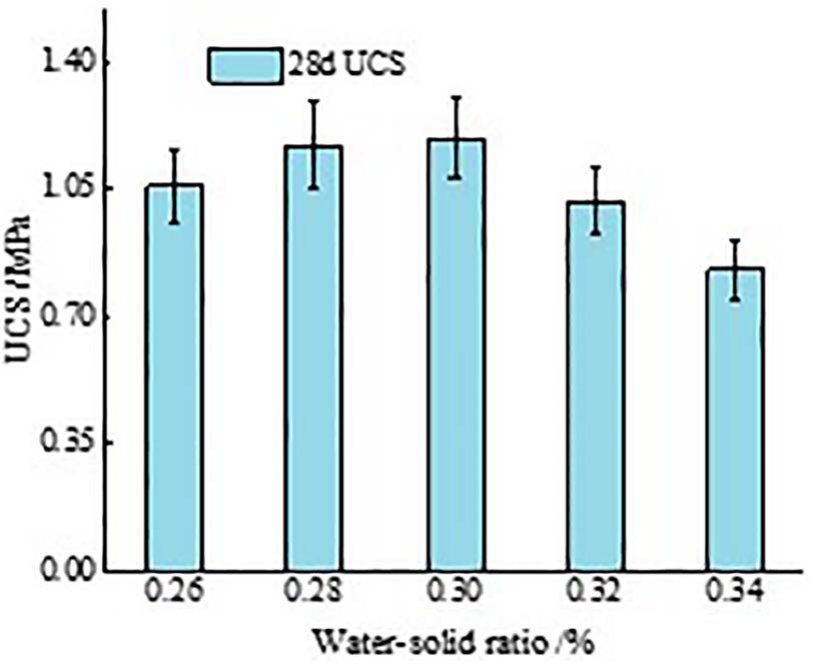
Compressive strength of samples with different water-solid ratios.

The unconfined compressive strength of GFSS with different water-solid ratios was tested, and the test results are shown in Fig 13.

**Fig 14:**
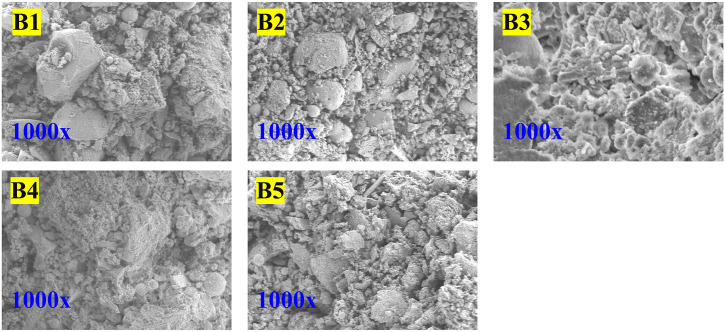
Microscopic morphology of samples with different water-solid ratios.

As can be seen from Fig 13, with the increase of the water-solid ratio, the compressive strength of GFSS shows a tendency to increase first and then decrease. When the water-solid ratio is 0.30, the compressive strength of the sample reaches a local maximum of 1.19 MPa. When the water-solid ratio is less than 0.30, the moisture in the slurry is insufficient, the dissolution of fly ash and slag is limited, the amount of geopolymer gel is generated is less, and the strength of the material is lower. Based on Fig 14, it can be seen that when the water-solid ratio increases to 0.30, the moisture content is sufficient but not too much, the reaction of the geopolymer is fully carried out, and a large amount of C-A-S-H and N-A-S-H is formed, thereby forming a dense microstructure and reaching the highest compressive strength. When the water-solid ratio is greater than 0.30, there is too much water in the slurry, diluting the concentration of the alkali trigger, reducing the reaction rate and gel generation efficiency, resulting in a decrease in strength. At the same time, when the water-solid ratio is too low, the pores between the solid particles cannot be fully filled by the gel, resulting in poor density of the material and the generated geopolymer gel cannot form a continuous gel network, so the strength of GFSS is lower. When the hydro-solid ratio is around 0.30, the generated gel forms a continuous and dense three-dimensional network structure, providing good compressive resistance to the material. When the water-solid ratio is too high, the concentration of reactants in the solution decreases, and the generated gel is sparse and dispersed, unable to support the particle framework. Evaporation after 28 days will leave a large number of capillary pores, significantly reducing the density of the sample, resulting in a decrease in compressive strength.

**Fig 15:**
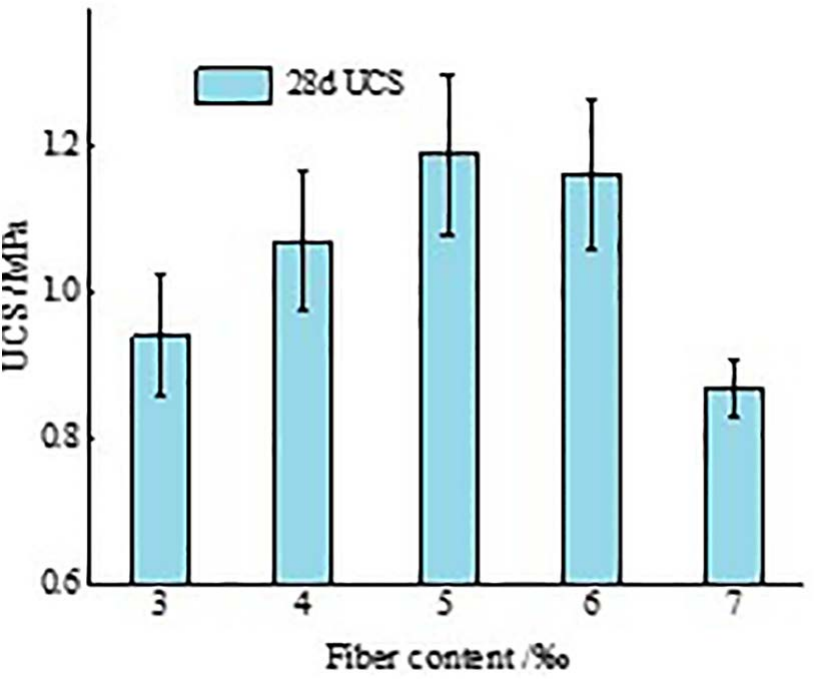
Compressive strength of samples with different fiber content.

Unbounded compressive strength test was performed on GFSS with different fiber doping amounts, and the test results were shown in Fig 15.

**Fig 16:**
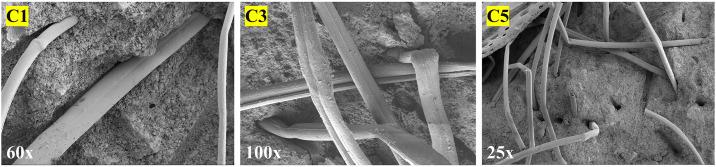
Microscopic morphology of samples with different fiber content.

As can be seen from Fig 15, with the increase of fiber dosage, the compressive strength of GFSS shows a tendency to increase first and then decrease. When the fiber dosage is 5‰, the compressive strength of the sample reaches the local maximum, which is 26.59% higher than when the fiber dosage is 3‰. In GFSS, when the fiber doping is small, the fibers can be distributed more evenly in the matrix, giving full play to its reinforcement effect. The interface between the matrix and the fiber is better combined and can jointly resist external pressure. Combined with Fig 16, the proper amount of fiber can overlap inside the geopolymer matrix to form a stable three-dimensional spatial reinforcement network, which significantly improves the structural integrity and anti-cracking deformation ability of the solidified soil. On the one hand, the crack bridging effect of fiber can effectively restrain the initiation of micro-cracks and the continuous expansion of macro-cracks; On the other hand, fiber can improve the stress state of matrix through stress transmission and load dispersion and then improve the compressive properties of composites macroscopically. This reinforcement mechanism is basically consistent with the relevant research conclusions of existing fiber-modified cementitious materials [14,30].

However, the higher the fiber content is, the better. When the fiber content is too high, the fibers are prone to problems such as winding, agglomeration and uneven dispersion during the mixing and curing of the slurry. Uneven fiber distribution will introduce many pores, interface defects and structural weak areas in the matrix, destroy the compactness and structural continuity of the solidified soil matrix, produce obvious deterioration effect, and finally cause a significant decline in compressive strength. This threshold effect of fiber content has also been widely confirmed in the study of similar fiber-modified geopolymers and solidified soil [15,31].

**Fig 17:**
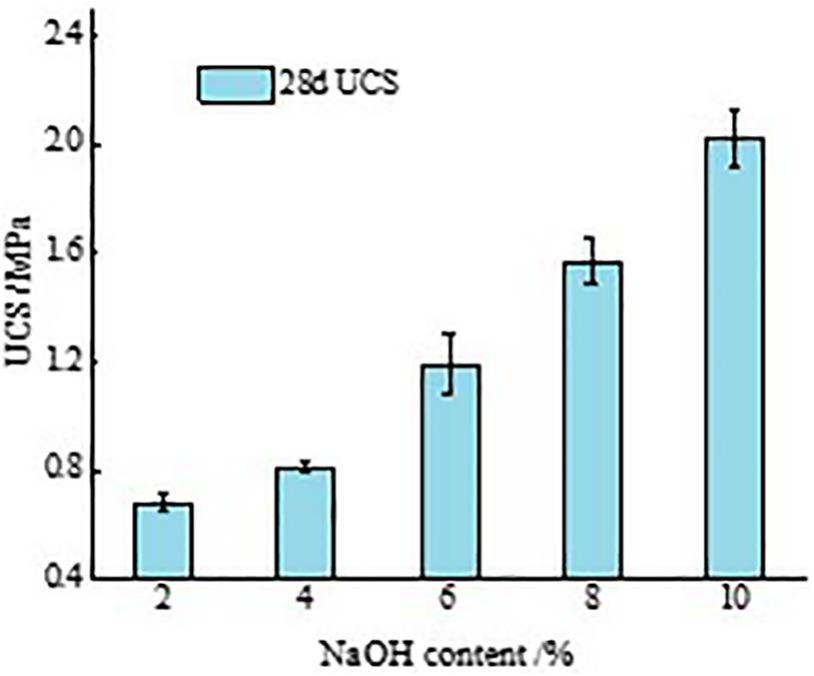
Compressive strength of samples with different sodium hydroxide contentcontent.

Unbounded compressive strength test was performed on GFSS with different NaOH dosages, and the test results were shown in Fig 17.

**Fig 18:**
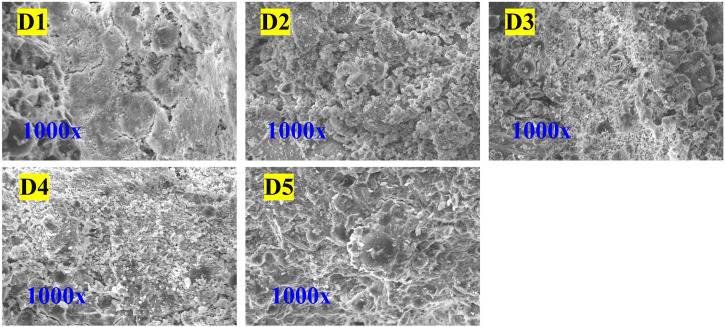
Microscopic morphology of samples with different sodium hydroxide content.

As can be seen from Fig 17, as the NaOH dosage increases, the compressive strength of the GFSS sample 28d gradually increases. When the NaOH dosage is 10%, the compressive strength of the sample reaches a local maximum of 2.02MPa, which is about three times larger than when the NaOH dosage is 2%. The curing process of GFSS depends on the geopolymer reaction. The NaOH solution can dissolve the active silica and aluminum trioxide in the raw material to form monomeric silicates and aluminate. As the NaOH dosage increases, the dissolution rate of the active components increases, providing more raw materials for the geopolymer reaction and promoting the formation of C-A-S-H and N-A-S-H colloids. Based on Fig 18, as the NaOH concentration increases, the geopolymer reaction becomes more sufficient, the number of gels generated increases, and the network structure becomes denser, which enhances the compressive strength of the cured soil.

In practical geotechnical engineering application, the material cost and environmental impact of soil curing agent are the key indicators to evaluate its popularization feasibility. In terms of economic feasibility, taking the treatment of 1-ton (1000 kg) aeolian soil as an example, the actual consumption of NaOH is about 16 kg when the maximum dosage ratio (16% cementitious material, in which NaOH activator accounts for 10% of cementitious material) is adopted in this study. Referring to the domestic average market price of industrial sodium hydroxide of about 3,000 ￥/t, the cost of NaOH treatment per ton of soil is about 48 ￥. It is worth noting that the remaining 90% of the cementing materials in the stabilizer system are bulk industrial solid wastes such as slag and fly ash, and the acquisition cost of the materials themselves is extremely low (usually only the transportation cost is considered). In contrast, if the traditional ordinary Portland cement is used for curing treatment (assuming that the conventional dosage is 15% and the unit price of cement is about 300 ~ 400 ￥/t), the material cost per ton of soil is about 45 ~ 60 ￥. Considering comprehensively, the all-solid waste geopolymer stabilizer proposed in this study is in the same order of magnitude as the traditional cement solidification technology in terms of total material cost, and it has good economic competitiveness in large-scale engineering application because it can obtain the support of potential industrial waste consumption policy.In terms of environmental safety and ecological benefits. The doped NaOH did not remain in the soil for a long time in the form of free strong alkali. As an efficient activator, NaOH is mainly used to break the Si-O and Al-O bonds in the glass structure of slag and fly ash. With the progress of geopolymer polycondensation, OH and Na in the system are consumed in large quantities and finally are stably solidified and physically wrapped in the generated three-dimensional inorganic polymer networks (such as N-A-S-H and C-A-S-H dense gels), thus greatly reducing the leaching alkalinity of solidified soil and the risk of environmental seepage. From the macroscopic ecological benefits, the solidification scheme completely replaces the traditional portland cement with high energy consumption and high CO emission by using industrial solid waste, which not only significantly reduces the carbon footprint of aeolian soil reinforcement project, but also provides an effective way for large-scale resource utilization of slag and fly ash, and realizes the green geotechnical engineering concept of “treating waste with waste.”

Although the influence of the mix ratio on the performance of GFSS was discussed from the perspective of macroscopic mechanics and qualitative microscopic morphology, the evolution of hydration products and pore structure could not be quantitatively revealed due to the limitation of SEM observation field. In the future research, X-ray diffraction (XRD), thermogravimetric analysis (TGA), mercury intrusion porosimetry (MIP) and other characterization methods will be systematically introduced to quantitatively analyze the phase change law and pore distribution characteristics of geopolymers under different curing agent content and water content, so as to construct a more rigorous ' microstructure-macroscopic performance ' structure-activity relationship.

## 5 Conclusions

(1) The flow spread of GFSS is controlled by multiple physical-chemical effects. It increases significantly with the increase of water-solid ratio, and decreases with the increase of cementing material, NaOH and fiber content. Increasing the water-solid ratio increases the free water in the system and effectively lubricates the solid particles; Adding cementitious materials or NaOH will accelerate depolymerization and geo-polymerization, consume free water and quickly increase the viscosity of slurry; The fibers form a network structure inside the slurry, which increases the macro sliding resistance.
(2) The GFSS slurry shows significant shear thinning and stress overshoot characteristics, and the rheological behavior conforms to the Bingham model. The shear stress and yield stress increase significantly with the increase of cementitious material, NaOH and fiber content, and decrease with the increase of water-solid ratio. The internal mechanism shows that the cementitious material and NaOH accelerate the geological polymerization and network crosslinking and consume free water; the fiber constructs a three-dimensional shear skeleton; the increase of water-solid ratio thickened the lubricating water film and effectively reduced the friction and cohesion between particles.
(3) The compressive strength of GFSS continues to increase with the increase of cementitious materials and NaOH content, which is due to the accelerated dissolution of Si / Al in high alkali environment, and a large amount of C-A-S-H gel is generated to fill the pores and consolidate the skeleton. There is a significant threshold effect between water-solid ratio and fiber content). An appropriate amount can ensure sufficient reaction and form a spatial bridging network, while an excessive amount can lead to capillary water pore formation and fiber agglomeration defects, respectively, causing strength degradation.

## Supporting information

S1 Data Data. (ZIP)
