# Supplementary material for: Rheological properties and compressive properties of alkali-activated slag-fly ash geopolymer fluid solidified soil
Source: PLoS One. 2026 Jul 10;21(7):e0350229. doi: 10.1371/journal.pone.0350229 (PMC13353964; doi:10.1371/journal.pone.0350229)
Supplement: S1 Data — (ZIP) [file pone.0350229.s001.zip › Table data.docx]

Table 1 Physical Properties of Polypropylene Fiber

| Fibre length /mm | Density/（g·cm^-3^） | Tensile strength/MPa | Elastic modulus/GPa | Melting point/℃ | Ignition point/℃ | Fracture elongation /% | Dispersibility |
| --- | --- | --- | --- | --- | --- | --- | --- |
| 9 | 0.91 | ≥350 | ≥3.5 | ＞160 | 590 | ≥10 | Fabulous |

Table 2 Chemical composition of raw materials

| Material | CaO | Fe_2_O_3_ | Al_2_O_3_ | SiO_2_ | MgO | SO_3_ | K_2_O | Na_2_O |
| --- | --- | --- | --- | --- | --- | --- | --- | --- |
| Slag | 42.15 | 0.41 | 12.88 | 33.51 | 5.98 | - | 0.21 | 0.28 |
| Flyash | 4.15 | 3.65 | 24.99 | 59.11 | 0.42 | 0.35 | 1.15 | 0.99 |

Table 3 Test plan

| Number | Cementitious material content | Water-solid ratio | Fiber content /‰ | NaOH content /% |
| --- | --- | --- | --- | --- |
| A1 | 8% | 0.30 | 5 | 4 |
| A2 | 10% |  |  |  |
| A3 | 12% |  |  |  |
| A4 | 14% |  |  |  |
| A5 | 16% |  |  |  |
| B1 | 12% | 0.26 | 5 | 4 |
| B2 |  | 0.28 |  |  |
| B3 |  | 0.30 |  |  |
| B4 |  | 0.32 |  |  |
| B5 |  | 0.34 |  |  |
| C1 | 12% | 0.30 | 3 | 4 |
| C2 |  |  | 4 |  |
| C3 |  |  | 5 |  |
| C4 |  |  | 6 |  |
| C5 |  |  | 7 |  |
| D1 | 12% | 0.30 | 5 | 2 |
| D2 |  |  |  | 4 |
| D3 |  |  |  | 6 |
| D4 |  |  |  | 8 |
| D5 |  |  |  | 10 |

Table 4 Fitting results of Bingham model under different factors

| Test number | Yield stress $\tau_{0}$/pa | Plastic viscosity /pa·s | Fitting equation | R2 |
| --- | --- | --- | --- | --- |
| 1 | 57.50 | 0.39 | $\tau=57.50+0.39\dot{}$ | 0.93 |
| 2 | 62.61 | 0.09 | $\tau=62.61+0.09\dot{}$ | 0.76 |
| 3 | 92.49 | 0.84 | $\tau=92.49+0.84\dot{}$ | 0.94 |
| 4 | 110.59 | 1.37 | $\tau=110.59+1.37\dot{}$ | 0.95 |
| 5 | 186.82 | 0.48 | $\tau=186.82+0.48\dot{}$ | 0.95 |
| 6 | 261.07 | 0.60 | $\tau=261.07+0.60\dot{}$ | 0.97 |
| 7 | 151.24 | 0.83 | $\tau=151.24+0.83\dot{}$ | 0.97 |
| 8 | 92.49 | 0.84 | $\tau=92.49+0.84\dot{}$ | 0.94 |
| 9 | 40.17 | 0.31 | $\tau=40.17+0.31\dot{}$ | 0.98 |
| 10 | 34.70 | 0.31 | $\tau=34.70+0.31$ | 0.79 |
| 11 | 29.82 | 0.20 | $\tau=29.82+0.20\dot{}$ | 0.89 |
| 12 | 49.71 | 0.84 | $\tau=49.71+0.84\dot{}$ | 0.83 |
| 13 | 92.50 | 0.84 | $\tau=92.50+0.84\dot{}$ | 0.94 |
| 14 | 130.56 | 0.86 | $\tau=130.56+0.86\dot{}$ | 0.93 |
| 15 | 185.24 | 0.96 | $\tau=185.24+0.96\dot{}$ | 0.94 |
| 16 | 77.33 | 0.30 | $\tau=77.33+0.30\dot{}$ | 0.97 |
| 17 | 92.50 | 0.84 | $\tau=92.50+0.84\dot{}$ | 0.94 |
| 18 | 133.13 | 0.30 | $\tau=133.13+0.30$ | 0.79 |
| 19 | 143.88 | 0.78 | $\tau=143.88+0.78\dot{}$ | 0.91 |
| 20 | 257.92 | 0.82 | $\tau=257.92+0.82\dot{}$ | 0.85 |
